# Theory of skyrmions in bilayer systems

**DOI:** 10.1038/srep42645

**Published:** 2017-02-15

**Authors:** Wataru Koshibae, Naoto Nagaosa

**Affiliations:** 1 RIKEN Center for Emergent Matter Science (CEMS), Wako, Saitama 351-0198, Japan; 2 Department of Applied Physics, The University of Tokyo, 7-3-1, Hongo, Bunkyo-ku, Tokyo 113-8656, Japan

## Abstract

Skyrmion is an emergent particle consisting of many spins in magnets, and has many nontrivial features such as (i) nano-scale size, (ii) topological stability, (iii) gyrodynamics, and (iv) highly efficient spin transfer torque, which make skyrmions the promising candidate for the magnetic devices. Earlier works were focusing on the bulk or thin film of Dzyaloshinskii-Moriya (DM) magnets, while recent advances are focusing on the skyrmions induced by the interfaces. Therefore, the superstructures naturally leads to the interacting skyrmions on different interfaces, which has unique dynamics compared with those on the same interface. Here we theoretically study the two skyrmions on bilayer systems employing micromagnetic simulations as well as the analysis based on Thiele equation, revealing the reaction between them such as the collision and bound state formation. The dynamics depends sensitively on the sign of DM interactions, i.e., helicities, and skyrmion numbers of two skyrmions, which can be well described by Thiele equation. Furthermore, we have found the colossal spin-transfer-torque effect of bound skyrmion pair on antiferromagnetically coupled bilayer systems.

Skyrmion first proposed in nuclear physics as a model for mesons and baryons^1^ attracts intensive attention in condensed matter physics especially since its discovery in chiral magnets^2^,^3^,^4^,^5^,^6^,^7^,^8^,^9^,^10^,^11^,^12^,^13^,^14^,^15^,^16^,^17^,^18^,^19^,^20^,^21^,^22^,^23^,^24^,^25^,^26^,^27^,^28^,^29^,^30^ with Dzyaloshinskii-Moriya (DM) spin-orbit interaction^31^,^32^. It has the swirling magnetic configuration, where the normalized magnetic moment ***n***_***r***_ at the spatial coordinate ***r*** points all the possible directions as ***r*** covers the whole two-dimensional plane, and is characterized by the topological skyrmion number *N*_*sk*_,


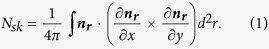


which counts this solid angle in unit of 4*π*. Here, even though its size of the order of 10 nm~100 nm is much smaller than the magnetic bubbles induced by the dipolar interaction, the magnetic moments ***n***_***r***_ change slowly in space and are described as the continuous function of ***r***. The skyrmion number *N*_*sk*_ cannot change for the continuous deformation of this magnetic configuration, and hence skyrmion is protected by topology and stable. Another important property of skyrmion comes also from the solid angle and *N*_*sk*_, i.e., the gyrodynamics^2^,^33^. Namely, the equation of motion for the center of mass has the similar form to that of a charged particle under magnetic field, and is subject to the efficient spin transfer torque (STT) effect^26^. This fact makes the dynamics of skyrmion very unique and distinct from that of domain walls in ferromagnets, and leads to the very small threshold for the current-driven motion^13^,^22^. Skyrmion is basically the two-dimensional structure, and realized as the rod-type string-like object in three-dimensional crystal along the external magnetic field^9^. In the two-dimensional systems such as the thin films, it acts as a zero-dimensional particle as long as the thickness is smaller than the size of the skyrmion radius^12^. Recent advances are focusing on the skyrmions induced by the interfaces. In this case, the inversion symmetry is broken at the interface even though the constitute materials are centrosymmetric. By the artificial superstructures including mutilayers^34^,^35^,^36^,^37^,^38^,^39^,^40^,^41^, one can control the interlayer interactions, which offers much richer possibilities than the thin film of single crystals where the interaction along the *z*-direction is dominated by the strong ferromagnetic interaction.

The interaction between the two skyrmions on the same two-dimensional plane is the short-range repulsion, and no bound state is formed. However, when the two skyrmions are on the separate planes and can overlap, there are variety of possibilities depending on the interlayer exchange interaction between the layers, and the signs of the DM interactions.

In this paper, we investigate theoretically the dynamics of two skyrmions in this setup in terms of the numerical simulation of the Landau-Lifshitz-Gilbert (LLG) equation and also the Thiele equation for each skyrmion. It is found that the latter is an excellent approximation, and correctly describe the reaction between the two skyrmions such as collision and bound state formation. Giant spin transfer torque effect of a bound state is also revealed.

## Results

### Model

**Figure 1:**
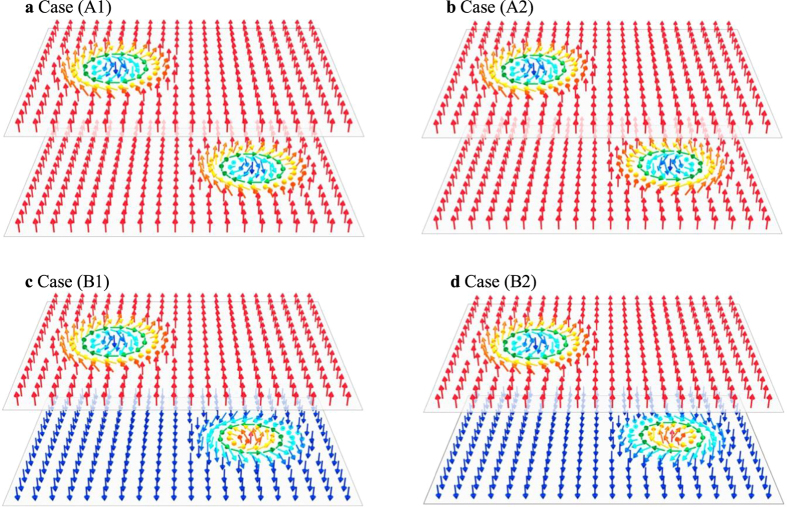
Skyrmions in bilayer system. (**a**) The skyrmions on each layer have the same skyrmion number *N*_*sk*,1_ = *N*_*sk*,2_ due to the ferromagnetic interlayer coupling *J*_inter_ > 0, and those helicity are the same as each other. (**b**) The helicity of the skyrmion on layer 2 is opposite to that in the case (A1). (**c**) Compared to the case (A1), the magnetic texture on layer 2 is totally reversed topologically and hence *N*_*sk*,1_ = −*N*_*sk*,2_, due to the antiferromagnetic interlayer coupling *J*_inter_ < 0. In this case, we do not apply the external magnetic field and instead the magnetic anisotropy *K* is introduced to stabilize skyrmions. (**d**) The helicity of the skyrmion on layer 2 is opposite to that in the case (B1).

The model Hamiltonian for the bilayer skyrmion magnet is given by


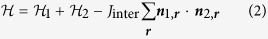


and


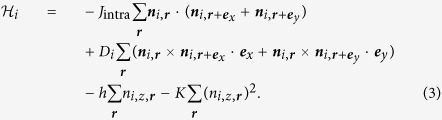


(See also the Supplementary Information). Here, 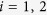 is the layer index. The basis {***e***_*x*_, ***e***_*y*_} defines the two-dimensional square lattice for each layer, and ***n***_*i*,***r***_ is the normalized magnetic moment at the site (*i, **r***). We take the lattice constant *a* of a layer as the unit of length. The Hamiltonian 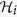 involves the ferromagnetic interaction *J*_intra_, the uniaxial anisotropy *K* and the layer dependent DM interaction *D*_*i*_. The layers 1 and 2 are coupled by the Heisenberg interaction *J*_inter_. There are 4 cases depending on the signs of *J*_inter_ and *D*_*i*_ (see Fig. 1), i.e., (A1) The ferromagnetic *J*_inter_ > 0, and the same helicity (DM interaction) *D*_1_ = *D*_2_, (A2) The ferromagnetic *J*_inter_ > 0, and the opposite helicity (DM interaction) *D*_1_ = −*D*_2_, (B1) The antiferromagnetic *J*_inter_ < 0, and the same helicity (DM interaction) *D*_1_ = *D*_2_, and (B2) The antiferromagnetic *J*_inter_ < 0, and the opposite helicity (DM interaction) *D*_1_ = −*D*_2_. As shown below, the dynamics is quite different among these 4 cases.

### Potential energy between two skyrmions on different layers

**Figure 2:**
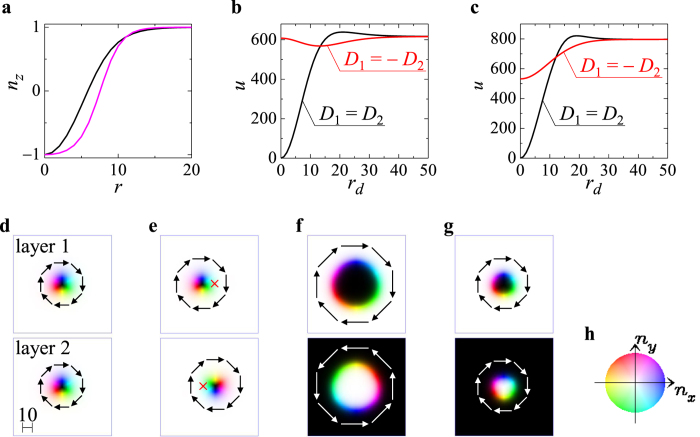
Skyrmions in bilayer system. (**a**) The profile of the *n*_*z*_ component of the skyrmion solution 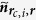 (see text) as a function of the radius *r* = |***r*** − ***r***_*c*,*i*_| where the single skyrmion is on layer *i* and the other layer is a perfect ferromagnetic state along *n*_*z*_ axis. The black (purple) curve is for the cases (A1) and (A2) (the cases (B1) and (B2)). (**b**) The normalized potential 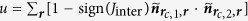 as a function of *r*_*d*_ = |***r***_*c*,1_ − ***r***_*c*,2_| for *D*_1_ = *D*_2_ (black) and *D*_1_ = −*D*_2_ (red) corresponding to the cases (A1) and (A2), i.e., the ferromagnetic interlayer coupling. The interaction energy between the two skyrmions on different layers is given by *U* = |*J*_inter_| *u*. (**c**) The same as (**b**) but for the cases (B1) and (B2), i.e., the antiferromagnetic interlayer coupling. (See the section Methods for the detail of numerical conditions). In the figures (**d**–**g**), the magnetic textures for the cases (A1), (A2), (B1) and (B2) which minimize the total energy are shown, respectively. The length scale is shown in the lower panel of (**d**). The arrows indicate the winding direction of the in-plane components of the magnetic moments. The color code (**h**) is used to express the magnetic textures ***n***_*i*,***r***_, where the color represents the direction of in-plane magnetic moments and the brightness does the out-of-plane magnetic moments, e.g., white and black correspond to *n*_*i*,*z*,***r***_ = +1 and =−1, respectively. In figure (**e**), the red symbols × on layer 1 and 2 represent the center-of-mass of the skyrmion on layer 2 and 1, respectively.

In the bilayer systems with the cases (A1), (A2), (B1), and (B2) defined above, let us first discuss the interaction between skyrmions, i.e., a skyrmion is on layer 1 and another one is on layer 2. When the two skyrmions are sufficiently separated with each other, the skyrmion solution 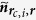 with the center-of-mass 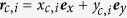 on layer *i* is the same as that where the other layer is the perfect ferromagnetic state. Figure 2a shows the *n*_*z*_ component of 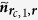 as a function of the radius measured from the center 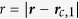, i.e., the black (purple) curve is for the cases (A1) and (A2) (the cases (B1) and (B2)). The details of numerical condition are described in the section Methods. Here, we chose the parameter set where the skyrmion sizes corresponding to the black and purple curves in Fig. 2a are similar to each other. In the case (A1), 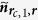 and 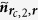 are the same except for the spatial positions ***r***_*c*,1_ and ***r***_*c*,2_. Compared with this, the in-plane magnetic moments of 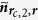 are reversed in the case (A2). In the case (B1), 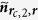 is given by the totally reversed magnetic moments of 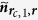 except for the spatial positions ***r***_*c*,1_ and ***r***_*c*,2_. Compared with the case (B1), the in-plane magnetic moments of 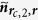 are reversed in the case (B2). In the case that 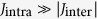, which we focus in most parts of the present study, we define the “normalized potential” 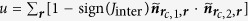 to evaluate the skyrmion interaction. In Fig. 2b,c the black and red curves show the *r*_*d*_ dependence of *u* for the cases (A1) and (A2) ((B1) and (B2)), respectively. In case (A1), there exists an energy barrier and hence the repulsive force acts for the large *r*_*d*_ while it turns into attractive for small *r*_*d*_ (black curve in Fig. 2b). In case (A2), on the other hand, the potential has the minimum at *r*_*d*_ ≠ 0, i.e., the interaction is attractive for the large *r*_*d*_ and is repulsive when *r*_*d*_ is small enough (red curve in Fig. 2b). In the case (B1), the behavior of *u* is similar to that in (A1), but the potential maximum is slightly shifted to shorter *r*_*d*_ (black curve in Fig. 2c). The potential is always attractive in the case of (B2) although weaker than cases of (A1) and (B1) (red curve in Fig. 2c).

Because of the interaction described by Fig. 2b,c, the skyrmions form the bound states as shown in Fig. 2d–g, in the cases (A1), (A2), (B1), and (B2), respectively. In particular, we find that *r*_*d*_ = 0 for the bound state in the cases (A1), (B1), and (B2), but *r*_*d*_ ≠ 0 in the case (A2).

In the above consideration on the normalized potential *u*, we neglect the deformation of the skyrmions. As seen in Fig. 2d–g, the skyrmions are relaxed further, in particular, in the case (B1), the skyrmion size is enlarged as shown in Fig. 2f. The role of the deformation of the skyrmions is further discussed in the following sections.

### Dynamics of skyrmions

The dynamics of the magnetic texture is described by the LLG equation,


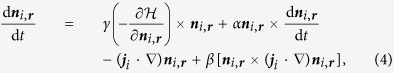


where *γ* is for the gyromagnetic ratio, and *α* denotes the Gilbert damping constant. The last two terms represent the STT effect by the (spin-polarized) electric current density ***j***_*i*_, and the nonadiabatic effect with the constant *β*.

When we neglect the dynamical deformation of the skyrmions, the LLG equation Eq. (4) is reduced to the Thiele equation as the equation of motion of the skyrmion on *i*-th layer,


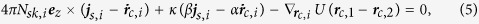


where 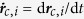 and ***e***_*z*_ is the unit vector perpendicular to the *xy*-plane spanned by {***e***_*x*_, ***e***_*y*_}. The external spin current due to the spin polarized electron current is denoted by ***j***_*s*,*i*_ (which sign is opposite to that of the electric current ***j***_*i*_ in the positively polarized metallic magnet). A dimensionless constant *κ* (=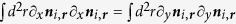 in this study) is several times of *π*^26^. In the last term, *U* = |*J*_inter_| *u* and 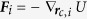 represents the force acting on the skyrmion.

**Figure 3:**
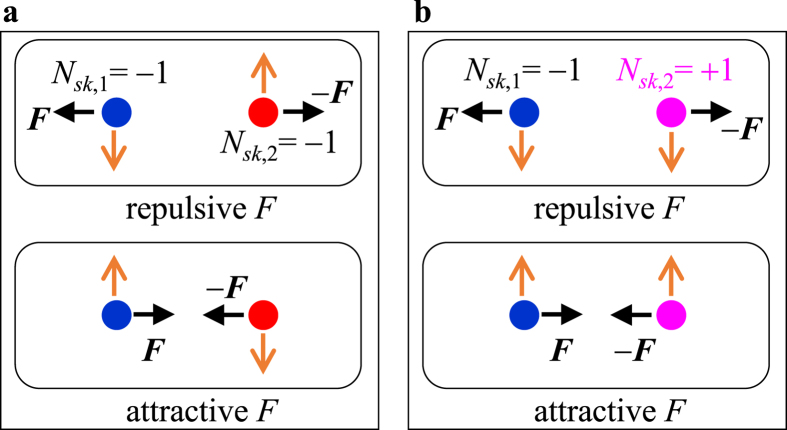
Gyrodynamics of skyrmions. (**a**) Velocity (orange arrow) of each skyrmion is induced by the force (black arrow) acting on it when *N*_*sk*,1_ = *N*_*sk*,2_, i.e., the cases (A1) and (A2). (**b**) The same as (**a**) but for the cases (B1) and (B2) where *N*_*sk*,1_ = −*N*_*sk*,2_.

The first term of the Thiele equation Eq. (5) determines the gyrodynamics of the skyrmion, and its effect is schematically represented in Fig. 3a,b for the cases (A1) and (A2) ((B1) and (B2)): Here, the black arrow indicates the direction of the force ***F*** = ***F***_1_ = −***F***_2_, and the blue and red dots in Fig. 3a are the skyrmion on layer 1 and that on layer 2, respectively. In the cases (A1) and (A2), 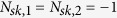. Therefore, the gyrodynamics gives a mutual rotation in the counterclockwise (clockwise) direction for the repulsive (attractive) interaction ***F***. In the cases (B1) and (B2), on layer 2, the sign of the skyrmion number is opposite, i.e., 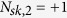 as represented by the purple dot in Fig. 3b and hence the parallel motion instead of the rotational motion is induced.

**Figure 4:**
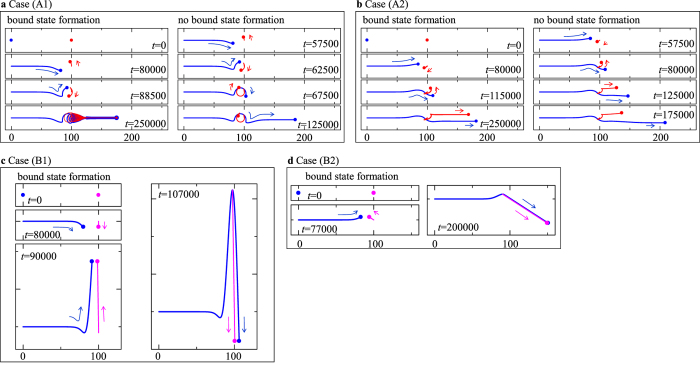
Skyrmion dynamics described by Thiele equation Eq. (5). The figures (**a**–**d**) show the dynamics in the cases (A1), (A2), (B1) and (B2), respectively, using the potential in Fig. 2b,c (see text). The blue dot indicate the skyrmion on layer 1. The red dot represents the skyrmion on layer 2 for the cases (A1) and (A2). The purple dot is also for the skyrmion on layer 2, but in the cases (B1) and (B2). On layer 1, the current ***j***_*s*,1_ = *j*_*s*,1_***e***_*x*_ is applied to make the skyrmion (blue dot) on layer 1 collide with the other skyrmion (red or purple dot) on layer 2 (see text). The trajectory of the skyrmion on layer 1 is shown by the blue curves. The red and purple curves are the trajectories of the skyrmion on layer 2, respectively.

From the skyrmion solutions and *U* = |*J*_inter_| *u* shown in Fig. 2a–c, one can derive the Thiele equation Eq. (5) and find the variety of the skyrmion dynamics as shown in Fig. 4a–d. Below, the time *t* and the magnitude of ***j***_*s*,*i*_ are measured in units of 1/(*γ* *J*_intra_) and 2*eγ* *J*_intra_/*pa*^2^ (*p*: polarization of magnet), and the units are typically 1/(*γ* *J*_intra_) ~ 0.7 ps and 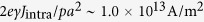, respectively, if we assume 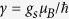 (*g*_*s*_: electron spin *g*-factor, *μ*_*B*_: Bohr magneton), *J*_intra_ ~ 10^−3^ eV, *p* = 0.2, and *a* = 5 Å.

Figure 4a,b show the current driven dynamics of the skyrmions in the cases (A1) and (A2), respectively, i.e., the blue and red dots are the skyrmions on layer 1 and 2, respectively, and those trajectories are represented by the blue and red lines, respectively. First, we focus on the dynamics where the skyrmions form a bound state (left panels of Fig. 4a,b): In the initial state, we put the skyrmions with a distance *r*_*d*_ = 100 at *t* = 0. On layer 1, we apply an STT effect by ***j***_*s*,1_ = *j*_*s*,1_***e***_*x*_ with *j*_*s*,1_ = 0.001 and drives the skyrmion (blue dot in Fig. 4a). Here, we use *β* = *α*(=0.01) to make the skyrmion (blue dot) on layer 1 collide with the other skyrmion (red dot) on layer 2. The skyrmion on layer 1 approaches the skyrmion on layer 2, and the skyrmions start to show a mutual rotation in counterclockwise direction. Later, the rotation direction changes, and finally the skyrmions form a bound state. The gyrodynamics is consistent with the force ***F*** driven by the normalized potential *u* shown by the black curve in Fig. 2b, namely, the potential is first repulsive and later turns into attractive.

The skyrmion bound state formation in the case (A2) is shown in the left panel of Fig. 4b. Because the magnitude of the force ***F*** is much smaller than that in the previous case (A1), it is difficult to form a bound state by the skyrmion collision for small *α*. Here, we use *α* = *β* = 1.0 and find the bound state formation for *j*_*s*,1_ = 0.001.

After the bound state is formed in the cases (A1) and (A2), the velocity of the bound skyrmions is 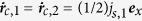 for *α* = *β*. In the steady state, 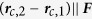 for the attractive force and hence we find the relation 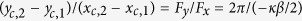 by the Thiele equation Eq. (5). We calculate *κ* using the procedure by ref. 26 (see also the section Methods), and the ratio 2*π*/(−*κβ*/2) is ~−0.86 from the present case. This is also seen by 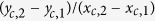 at *t* = 250000 in the left panel of Fig. 4b due to the unique structure of *u* shown by the red curve in Fig. 2b.

When the current *j*_*s*,1_ is larger than a critical value, the skyrmion bound state is not formed. The right panels of Fig. 4a,b show the examples with *j*_*s*,1_ = 0.0014 where the two skyrmions rotate around each other for a while but are separated afterward, i.e., the skyrmion on layer 2 is left behind.

In the cases (B1) and (B2), when the syrmion bound state is formed, we find


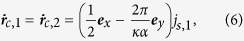


for *α* = *β*. The steady states of the bound skyrmions in these cases are shown in the right panels of Fig. 4c,d, i.e., at *t* = 107000 for the former one and at *t* = 200000 for the latter one. Before the bound state formation is completed in the case (B1), the skyrmion on layer 1 is running in a non-monotonic manner driven by the gyrodynamics, i.e., as shown in the panels at *t* = 80000 and at *t* = 90000 of Fig. 4c, the skyrmion is going in −***e***_*y*_ direction first, and later moves in +***e***_*y*_ direction. This is because of the change of the potential force from repulsive to attractive with decreasing *r*_*d*_ (see the black curve in Fig. 2c). Along with this non-monotonic dynamics, the skyrmion on layer 2 also shows the corresponding motion, i.e., it moves in −***e***_*y*_ direction first and later moves in +***e***_*y*_ direction. Finally the two skyrmions form a bound state and show the motion described by Eq. (6). In the case (B2), before the bound state formation, we only see the motion in +**e**_*y*_ direction as a result of the gyrodynamics. This is also explained by the normalized potential *u* shown in Fig. 2c (see the red curve).

### LLG dynamics in skyrmion collision

Next, we study the skyrmion dynamics by the LLG equation Eq. (4) to compare with that discussed above by the Thiele equation Eq. (5). Below we study the systems with periodic boundary condition to avoid the effects from the boundaries. (See the section Methods for the details of the numerical conditions).

**Figure 5:**
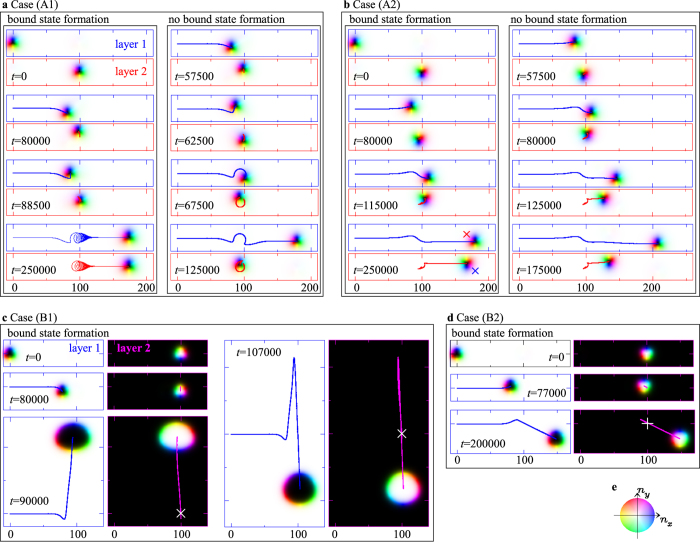
LLG dynamics of skyrmion collision. The parameter sets for the results shown in (**a**–**d**) correspond to the cases shown in Fig. 4a–d, respectively. The panels with blue frames show the magnetic textures on layer 1. In the figures (**a**–**d**), the magnetic textures on layer 2 are shown with red (purple) frames for the cases (A1) and (A2) ((B1) and (B2)). To represent the magnetic texture, the color code (e) is used. The trajectory of the skyrmion on layer 1 is shown by the blue curves. In the figures (**a**–**d**), the red (purple) curves are the trajectories of the skyrmion on layer 2 for the cases (A1) and (A2) ((B1) and (B2)). In the left panel of (**b**) at *t* = 250000, the red (blue) symbol × on layer 1 (layer 2) represents the center-of-mass of the skyrmion on layer 2 (layer 1). In figures (**c**,**d**), the white symbols × and + indicate the initial position of the skyrmion on layer 2.

Figure 5 shows the results of the LLG dynamics. By comparing Figs 4a,b,d and 5a,b,d, we find that the Thiele equation Eq. (5) well reproduces the LLG dynamics quantitatively. In particular, in the result shown in Fig. 5b, at the steady state after forming the bound state, the ratio 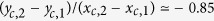 and it shows a good agreement with the result by the Thiele equation Eq. (5).

The agreement between Figs 4c and 5c is also good, but less than that between Figs 4a,b,d and 5a,b,d. We already see that the skyrmion size changes by the bound state formation in Fig. 2d–g and the largest change occurs in the case (B1) (Fig. 2f). The skyrmion size changes also occur along the time evolution shown in Fig. 5. The time-dependent deformation of the skyrmion causes not only the change in the parameter *κ* in Eq. (5) but also an emergence of the mass term beyond the Thiele equation^28^. This is responsible for the quantitative difference between the results by Thiele equation Eq. (5) and LLG equation Eq. (4).

**Figure 6:**
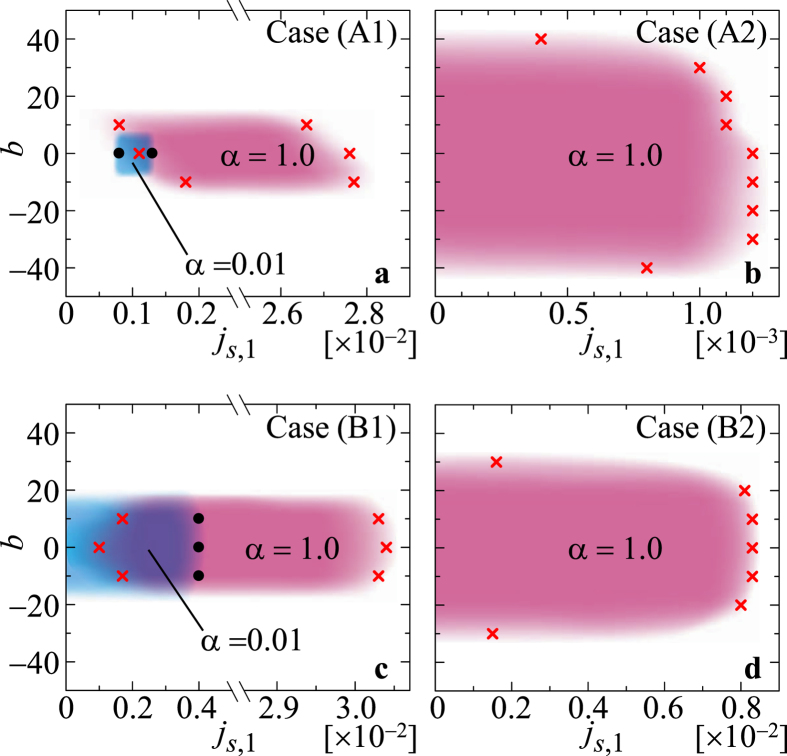
Parameter region of the bound state formation at the collision. (**a**) The black dots (red crosses) indicate the upper and lower critical values of *j*_*s*,1_ to form the skyrmion bound state for *α* = 0.01 (*α* = 1.0) in the case (A1). The critical values of *j*_*s*,1_ are examined at the impact parameters *b* = −50 ~ +50 in 10 increments. Figures (**b**–**d**) are the same as (**a**) but for the cases (A2), (B1) and (B2), respectively, and only the results for *α* = 1.0 are shown in (**b**,**d**).

We examine the bound state formation by the skyrmion collision in wide parameter region, *j*_*s*,1_ and *α* (=*β*). The normalized potential *u* shown in Fig. 2b,c tells us the spatial extent of the interaction between skyrmions. Therefore, we also examine the impact parameter *b* dependence for the bound state formation by the skyrmion collision. In the cases (A1) and (B1), because the potential curve *u* has a barrier shown in Fig. 2b,c, there exist upper and lower critical values of *j*_*s*,1_. We have examined the critical values of *j*_*s*,1_ at *b* = −50 ~ +50 in 10 increments, and summarize them in Fig. 6. For the case (B1) with *α* = 0.01, the magnitude of the lower critical current density is of order of 10^−5^ and is not plotted explicitly in Fig. 6c. The upper and lower critical values are larger for larger *α*, as shown in Fig. 6a,c. This is because, in the system with small damping, i.e., in the small *α* system, the system is easier to be excited and overcomes the energy barrier by the current *j*_*s*,1_. We also find the difference between the positive and negative values of *b* reflecting the vorticity and/or the gyrodynamics of the skyrmions as seen in Fig. 6. In the cases (A2) and (B2), corresponding to the potential curve *u* shown in Fig. 2b,c, we see the upper critical current density only. Because the potential force is much smaller than those in the cases (A1) and (B1), for *α* = 0.01, the upper critical value of *j*_*s*,1_ is order of 10^−4^ in the case (B2) and it is less than that in the case (A2). Therefore, only the results for *α* = 1.0 are shown in Fig. 6b,d.

## Discussion

### Colossal STT effect

As seen in Eq. (6), for the skyrmion bound state between the antiferromagnetically coupled layers, i.e., cases (B1) and (B2), an STT effect proportional to 1/*α* appears perpendicular to the external electric current. Here, we focus our attention on the case (B1) because of the efficient stability of the skyrmjon bound state (see black curve in Fig. 2c). Note that we do *not* apply the condition *α* = *β* in the following.

**Figure 7:**
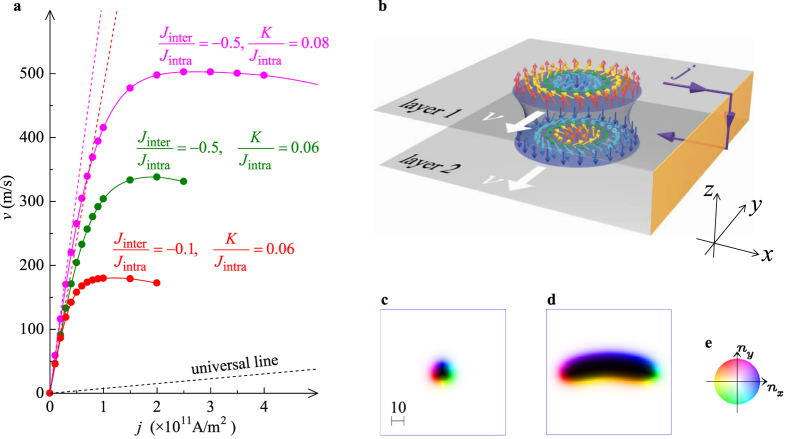
Colossal STT effect of bound pair of skyrmions in case (B1). (**a**) Current (*j*) – velocity (*v*) characteristics of skyrmion bound state (see text). The black broken line indicates the universal relation *v* = *j* by dimensionless *v* and *j*^26^. (**b**) Device structure. (**c**) Skyrmion structure on layer 1 for *j* = 0, *J*_inter_/*J*_intra_ = −0.1 and *K/J*_intra_ = 0.06 (corresponding to the red curve in (**a**)). A closeup at 100 × 100 area (see the length scale) is presented. (**d**) The same as (c) but for *j* = 2.0 × 10^11^ A/m^2^. To represent the magnetic textures (**c**,**d**), the color code (**e**) is used.

Figure 7a,b show the current (*j*)-velocity (*v*) characteristics with the enhancement of the STT effect calculated by the LLG equation Eq. (4), and a possible setup to realize this colossal STT effect, respectively. In this device, the electric current flows in opposite directions between layers 1 and 2. Therefore, the Thiele equation Eq. (5) gives


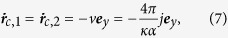


for 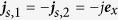.

The result Eq. (7) is similar to that shown in the previous studies for a skyrmion along the edge of the sample^27^,^29^. In that case, the skyrmion velocity has a maximum 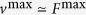 where *F*^max^ is the confining force of the skyrmion from the sample edge. The skyrmion confining force is ~*D*^2^/*J (J*: ferromagnetic interaction, *D*: DM interaction) and hence, in the previous study^29^, *v*^max^ ~ 20 m/s was estimated. We find that the maximum velocity seen in Fig. 7a is much larger than this value.

To enhance the skyrmion velocity, the key parameters are *K* and *J*_inter_: As seen in Fig. 7a, with increasing *j*, the skyrmion velocity given by the LLG simulation deviates from the purple and red broken lines given by Eq. (7) for corresponding *κ* values (see the section Methods). This is due to the deformation of the skyrmion. An example for the deformed skyrmion is shown in Fig. 7d. As discussed above, the deformation of the skyrmion causes some effects beyond the description in the Thiele equation Eq. (5). The skyrmions deformation increases with *j*, and finally the skyrmion bound state becomes unstable. In Fig. 7a, increase in *J*_inter_ stabilizes the skyrmion bound state and hence the skyrmion velocity is enhanced. In the case (B1), the skyrmion has a property of the magnetic domain enclosed by the circular domain wall (DW) with a width ~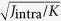, and the DM interaction gives a favorable helicity of the skyrmion. Because the tension of DW is proportional to ~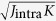, the skyrmion size shrinks with increasing *K*. We find that the shrinkage of the skyrmion brings about the enhancement of the factor 1/*κ*. In addition, the larger tension of the DW reduces the deformation of the skyrmion due to *j*. Therefore, large *J*_inter_ and *K* are favorable for the large STT effect, although too large *K* collapses the skyrmion.

Note that, in the STT effect discussed above, the value of *β* is totally irrelevant. This is in sharp contrast to the case that 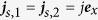, i.e., the currents flow in the same direction on the two layers. In this case, the Thiele equation Eq. (5) gives 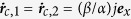. This was confirmed by LLG simulation.

### Validity of Thiele equation

The magnetic bubbles studied intensively in 70’s and 80’s aiming at the applications to magnetic memories are stabilized by the competition between the magnetic anisotropy *K* and the long-range magnetic dipolar interaction^33^,^42^,^43^,^44^,^45^. Usually the dipolar interaction is weaker than the DM interaction and hence the size of the bubble is much larger than the skyrmion stabilized by DM interaction. (Note that some of the magnetic bubbles are actually skyrmions). Dipolar interaction allows the degeneracy with respect to the helicities. Together with the large size, this degrees of freedom gives the flexibility of the shape of the magnetic bubble, and hence the analysis of the motion including the deformation becomes a rather complex issue. Actually, we have recently studied this problem, and have found that the internal force between the different parts of the bubble induces the center-of-mass motion as revealed by the simulation of LLG equation^45^. Although the qualitative understanding of this motion is possible in this case, the analytic method cannot offer the quantitative explanation. In sharp contrast to the case of these bubbles, the deformation of skyrmions stabilized by DM interaction is much suppressed, and the center-of-mass motion is described well by Thiele equation. Namely, they behave as independent point particles, and once one knows the interaction between skyrmions as in Fig. 2b,c, the motion can be predicted precisely by Thiele equation without the LLG equation. This remains true even when we increase the number of skyrmions, which offers a very powerful route to design the dynamics of many-body skyrmionic systems including multilayer systems and many-skyrmions in the same layer. Note that even for the skyrmions stabilized by the magnetic dipolar interaction, the larger anisotropy energy *K* reduces the internal deformation and the Thiele equation Eq. (5) works better. Such metallic magnets, therefore, are also the candidates for the table top experiments to realize our theoretical results.

### Interlayer interaction *J*_inter_

There are already several experimental works to manipulate the multi-layer magnetic systems^34^,^35^,^36^,^37^,^38^,^39^,^41^,^46^,^47^,^48^,^49^. The interlayer interaction *J*_inter_ can be controlled by the spacer between the skyrmion layers. When it is a nonmagnetic insulating material, the penetration of the wavefunctions and their overlap mediates the ferromagnetic exchange interaction. In this case, the ferromagnetic *J*_inter_, which decay exponentially with the thickness, results. When the spacer is metallic, the exchange interaction through the spacer oscillates with the wavenumber 2*k*_*F*_ with *k*_*F*_ being the Fermi wavenumber. In this case, the sign of *J*_inter_ changes as a function of thickness. In the case of spacer of antiferromagnetic magnetic insulator, the sign of *J*_inter_ shows even-odd effect as the number of layers of the spacer. These variety of materials offers the way to design and control *J*_inter_ in the realistic experimental setups.

## Conclusions

We have studied the interaction between the skyrmions and their dynamics in the bilayer systems, where the two layers are coupled by *J*_inter_. There are four cases depending on the sign of *J*_inter_ and the relative sign of the DM interactions on two layers. The interaction between the skyrmions as a function of the mutual distance is quite different among these four cases, which produces the rich variety of collision dynamics as revealed by the simulations of LLG equation. This complex behavior can be reproduced almost perfectly by Thiele equation quantitatively, which comes from the robustness of the skyrmion against the internal deformation. This means that large scale simulation of LLG equation is not needed, which can be replaced by the solution of Thiele equation regarding each skyrmion as a point particle. This fact indicates that Thiele equation offers a powerful way to design the skyrmion dynamics in many situations, which cannot be the case for the magnetic bubbles with larger size. We regard this as one of the advantage of skyrmions for applications, and will give an important guiding principle for skyrmionics employing many skyrmions.

## Methods

For the LLG dynamics in the cases (A1) and (A2), we use the numerical condition, {*J*_intra_ = 1.0, *D*_1_ = 0.2, *J*_inter_ = 0.004, *h* = 0.035, *K* = 0.0}, and each layer is 300 × 300 square lattice with the periodic boundary condition. The magnetic field *h* = 0.035 is slightly larger than the upper critical value 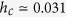 for the skyrmion crystal state in the single layer system^20^,^26^, so that the single skyrmion state is a metastable one for the small interlayer interaction *J*_inter_. In the cases (B1) and (B2), the parameter set {*J*_intra_ = 1.0, *D*_1_ = 0.2, *J*_inter_ = −0.004, *h* = 0.0, *K* = 0.05} is used except for the results shown in Fig. 7. The center-of-mass of the skyrmion is given by 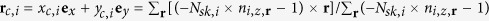.

Following the procedure by ref. 26, we numerically estimate the value *κ* by


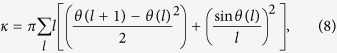


with cos *θ*(*l*) = *n*_*i*,*z*,***r***_. This equation Eq. (8) and *n*_*i*,*z*,***r***_ shown in Fig. 2a give 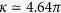, (6.17*π*) in the cases (A1) and (A2) ((B1) and (B2)). In the same way, for the results shown in Fig. 7a, we estimate 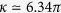, (4.85*π*) for *j* = 0 and *K/J*_intra_ = 0.06 (0.08) and plot the red (purple) broken line.

## Additional Information

**How to cite this article:** Koshibae, W. and Nagaosa, N. Theory of skyrmions in bilayer systems. *Sci. Rep.* **7**, 42645; doi: 10.1038/srep42645 (2017).

**Publisher's note:** Springer Nature remains neutral with regard to jurisdictional claims in published maps and institutional affiliations.

## Supplementary Material

Supplementary Video 1

Supplementary Video 2

Supplementary Video 3

Supplementary Video 4

Supplementary Video 5

Supplementary Video 6

Supplementary Information
