# Supplementary material for: Theory of skyrmions in bilayer systems
Source: Sci Rep. 2017 Feb 15;7:42645. doi: 10.1038/srep42645 (PMC5309827; doi:10.1038/srep42645)
Supplement: Supplementary Information [file srep42645-s7.pdf]

Theory of skyrmions in bilayer systems

Supplementary Information

Wataru Koshibae, Naoto Nagaosa

Effect of dipole-diploe interaction.

Here, we discuss the effect of dipole-dipole interaction for the large skyrmion-skyrmion distance r_d compared to the skyrmion size.

We suppose a single skyrmion is in layer 1 having a dimension $d\times L\times W$ (d: thickness, W: width, L:length) and, L and W are much larger than d and the skyrmion size.

The Bloch and Néel skyrmions are expressed to be

$$\boldsymbol{n}_{1,\boldsymbol{r}} = -\sin\theta_r \boldsymbol{e}_\phi + \cos\theta_r \boldsymbol{e}_z$$

and

$$\boldsymbol{n}_{1,\boldsymbol{r}} = -\sin\theta_r \boldsymbol{e}_r + \cos\theta_r \boldsymbol{e}_z ,$$

respectively, in a cylindrical coordinate system $\{\boldsymbol{e}_r, \boldsymbol{e}_\phi, \boldsymbol{e}_z\}$. Here, $r = |\boldsymbol{r}|$ is measured from the center of the skyrmion and the polar angle $\theta_r = \pi \to 0$ for $r = 0 \to \infty$. The magnetic charge ρ defined by $\rho_{1,\boldsymbol{r}} = -\,\mathrm{div}\,\boldsymbol{n}_{1,\boldsymbol{r}}$ is $\rho_{1,\boldsymbol{r}} = 0$ for the Bloch skyrmion, and $\rho_{1,\boldsymbol{r}}$ is finite but $\int \rho_{1,\boldsymbol{r}}\, d^2 r = 0$ for the Néel skyrmion. Therefore, the magnetic charge on the top and bottom surfaces of layer 1 defines a magnetic dipole by the skyrmion. On the top surface of layer 1, the difference of the magnetic charge from that of the perfect ferromagnetic state along $\boldsymbol{e}_z$ is given by $\cos\theta_r - 1$ and in total,

$$m_{sk} = \int d^2 r \left(\cos\theta_r - 1\right) = \int d^2 r \left\{-2\exp\left[-\left(r/r_0\right)^2\right]\right\} = -2\pi r_0^2$$

if we use a variational function $\cos\theta_r = 1 - \exp[-(r/r_0)^2]$ (Ref.[25]) for the z-component of the magnetic moment of the skyrmion. Here, r_0 represents the skyrmion size.

As a result, we find the potential energy E_{dipole} due to the dipole-dipole interaction between the skyrmion on layer 1 and that on layer 2 as,

$$E_{dipole} = \pm \frac{\left(d\, m_{sk}\right)^2}{4\pi} \frac{1}{\sqrt{r_d^2 + l^2}^{\,3}} \frac{r_d^2 - 2l^2}{r_d^2 + l^2} \approx \pm \frac{\left(d\, m_{sk}\right)^2}{4\pi} \frac{1}{r_d^3} \quad \text{for } r_d >> l ,$$

where l is the distance between the two layers and the sign + (–) is for the ferromagnetically (antiferromagnetically) coupled layers. The effect becomes weak if the exchange interactions, J_{intra}, J_{inter}, D_1, D_2 and so on, become strong. In particular, it is difficult to detect this effect for the room-temperature skyrmions.

Supplementary movies

A1.avi. Movie for the case “bound state formation” shown in Fig. 5a.

A1nob.avi. Movie for the case “no bound state formation” shown in Fig. 5a.

A2.avi. Movie for the case “bound state formation” shown in Fig. 5b.

A2nob.avi. Movie for the case “no bound state formation” shown in Fig. 5b.

B1.avi. Movie for the case “bound state formation” shown in Fig. 5c.

B2.avi. Movie for the case “bound state formation” shown in Fig. 5d.
